# Buffalo Academy of Medicine

**Published:** 1905-05

**Authors:** Franklin W. Barrows


### SOCIETY PROCEEDINGS.

### Buffalo Academy of Medicine.

*Section on Medicine, March 14, 1905.*

Reported by FRANKLIN W. BARROWS, M. D., Secretary.

THE regular meeting of the medical section was held Tuesday evening, March 14, 1905, at the academy rooms in the Public Library building. The meeting was called to order at 9 o'clock by the chairman, Dr. Allen A. Jones. The minutes of the last meeting were read and approved.

Dr. Elliott P. Joslin, of Boston, Mass., presented a paper, entitled,

### THE IMPROVEMENT IN THE TREATMENT OF DIABETES MELLITUS.

*(Author's Abstract.)*

The improvement in the treatment of diabetes mellitus is due chiefly to the substitution of facts for guess work. In the last ten years quantitative examination of the twenty-four hours' specimen of urine for sugar has become a routine practice; acetone and diacetic acid are always looked for, and by the determination of the urea or the nitrogen, an effort is made to control the amount of albumin in the diet.

The accurate determination of the sugar and starch in the diet of diabetics has also recently come into general use. This enables a comparison to be made between the carbohydrates in the diet and in the urine. It is thus known whether the patient excretes less carbohydrates than were contained in the diet, an equivalent amount, or more. The tolerance of the patient is thus determined, and a test is afforded by which all can determine the usefulness of any drug, food or method of treatment.

The aim in treatment is to increase the tolerance, and the first step to this end is to get the patient sugar-free. In most cases the partial withdrawal of carbohydrates from the diet brings this about. In others, a strict diet like the following, which is in use at the Boston City Hospital, is necessary: meat, fish, eggs, cheese, butter, coffee, tea, clear soups, gelatin, spinach, cabbage, cauliflower, string beans, lettuce, celery, tomatoes, radishes, asparagus, water cress, mushrooms, cucumbers, onions, olives, lemons, dandelions, pickles. Eat at least 3 eggs, 1 salad, 1 tablespoon olive oil, 3 vegetables and 2 ounces butter each day.

If these measures do not suffice various expedients can be tried. First, the reduction of the total quantity of food to an amount barely sufficient to maintain body weight. Should sugar still remain in the urine a second expedient—namely, the reduction of the albuminous food may cause it to disappear. A third resource is the vegetable day, in which coffee, tea, beef tea and diabetic vegetables are allowed. In the most severe cases starvation for twenty-four hours may be necessary.

Carbohydrates should be very gradually put back in the diet when their removal has been necessary to free the urine of sugar. Cream is the most useful carbohydrate food, and can slowly be added to the strict diet until half a pint is reached. Taking advantage of the fact that carbohydrates wholly in one form are better borne than a mixture of carbohydrates, it is next in most cases advisable to add small quantities of milk. Potatoes may be later tried, because they contain 20 per cent. of starch in contrast to the 60 per cent. in breads. The oatmeal cure is not applicable to most cases. It is well, however, to remember that a tablespoonful of cooked oatmeal contains but about one third of an ounce of sugar.

The discussion about milk, potato and oatmeal cures has given rise to the belief that the diabetic diet is less strict now than formerly. This is not true. The key note of treatment today remains the attainment of a sugar-free urine. The above diets, in certain cases, simply are means to this end.

The change from a general to a diabetic diet should be very gradual. Two thirds of a healthy man's food is sugar and starch, and it is not common sense to change the custom of years suddenly. The withdrawal of carbohydrates often leads to acid intoxication. In changing a diet it is a good rule to give a teaspoonful of bicarbonate of soda two or three times daily until the patient becomes accustomed to the new regime. Fat constitutes a great part of the diabetic diet. It should be added very slowly, and the taste disguised, lest the patient acquire repugnance for the same.

The treatment of coma lies in its prevention. It is a safe rule to proceed slowly in any dietetic change when the urine shows the ferric chloride reaction, and at the same time to give bicarbonate of soda. Good spirits, absence of restlessness or dulness and a slow diminution of the quantity of urine, as the carbohydrates in the diet are lowered, relieve the attendant of anxiety, even if diacetic acid is present. At the first untoward sign add to the diet a pint or more of milk, which should be taken in small quantities, and favor elimination of acids with liquids. Avoid acute dilatation of the stomach by caution in the administration of liquids. One teaspoonful of bicarbonate of soda may be given every half hour for three or four doses, then every hour, but soon every hour and one half to two hours for fear of vomiting. Avoid diarrhea. Enemata, as a rule, are safer than carthartics.

DISCUSSION.

Dr. Charles G. Stockton: One would like time for reflection before discussing this paper. The doctor has purposely omitted to mention special foods,—such as gluten. He has emphasised the importance of care in changing diet suddenly, which is, in a sense, a new idea to me. It is a suggestive idea,—that such a great oscillation as that from albumins to carbohydrates, for example, would greatly shock metabolism. The patient suffers from carbohydrate hunger for two reasons,—because he cannot utilise carbohydrates, and because the carbohydrates are withdrawn. When it is possible to omit the starvation day and vegetable day, we give the patient cream and then add potato. The patient craves a certain amount of starch. For that reason I have for years practised and taught that it is unwise to use bran and coarse cereals as a means of furnishing starch. There is no physiologic objection to using an equivalent of bread or potato. Some patients can use starch of one kind, but fail to use another kind; for example, they may use potatoes but not bread, or, bread but not oatmeal. My own experience in feeding milk has been unsatisfactory; so also with the sugars of fruits; both have brought back the sugar in the urine. I believe that drugs are useless in treating the disease, but not in promoting the general welfare of the patient. As to acid poisoning, I have seen patients approaching a state of coma who were improved by absolute rest, free elimination and alkalies. But I have never cured advanced coma by these methods. Other things than diet may precipitate coma. Fatigue is deadly in diabetes. I have had occasion to observe that patients on a restricted diet are often extraordinarily fatigued by very slight causes, such as a trifling physical effort or a mild mental excitement.

Dr. A. L. Benedict: Will the doctor please define diabetes? A rough clinical definition would be, according to my ideas, a failure to transform carbohydrates in the body, with compensatory breaking down of the proteids. I estimated at one time that the urine of diabetes would contain 100 grams, or more, of urea per day. Will Dr. Joslin please go into the question of urea in diabetes? Are the coloring matters of urine broken down by the tests for urea? As to medicines, strontium and the salicylates have, in my experience, given good results, though I do not know why.

Dr. Fridolin Thoma: Dr. Joslin's outlook for this disease, under dietetic treatment, is very hopeful. Von Naunyn, of Strasburg, states that one third of all cases investigated by him were hereditary. Many patients cannot be saved, especially between the ages of 18 and 22. He has discarded opium in treatment. I was induced to try the "Java plum," *Szygium Jambulanum,* after seeing it recommended by von Mering, of Halle. It seemed to help one of my cases. I advise exercise in the open air, as it leads to increased oxidation of sugar in the blood. A passing glycosuria is probably caused, at times, by a passing lesion of the pancreas.

Dr. Thomas G. Allen: In my experience ammonium salts are the best drugs. Ammonium salicylate, 5 or 10 grains, every three hours, and corrosive sublimate, 1-100 grain, have worked well. I prescribe also a moderate amount of exercise, and hot baths.

Dr. G. H. A. Clowes: Dr. Joslin's extremely interesting paper throws considerable light on several problems that have arisen in the course of our work in the laboratory. Starvation seems to be a most important factor in diabetes. Large quantities of proteids are prejudicial to the body. The suboxidation products of proteids are harmful. In analysis of the urine we should estimate ammonia as well as sugar and acetone. It is specially important to note the variation of individuals as to the kind and quantity of foods that they can take and assimilate. Potato contains, by percentage, nearly as much starch as does bread. But potato is better taken by diabetics than bread. So, also, milk sugar is easier to take than cane sugar. I have found by observation that the pancreatic extract of different individuals varies greatly in the power of digesting proteids, in some cases, digesting vegetable albumins better than animal albumins, and vice versa. It ought to be possible to slowly adapt the organs of digestion to any change of diet. Dr. Joslin's ingenious idea of giving a starvation diet, then a moderate vegetable diet and, finally, cream, forms the most rational plan of which I have ever heard for feeding diabetics. The addition of alkalies to raise the alkalinity of the blood helps in the removal of suboxidation products and those that interfere with oxidation.

Dr. Edmund E. Blaauw: I recollect that one great medical authority, Lépine, gives the inside of bread, never the crust, in diabetes.

Dr. Allen A. Jones: If we confine ourselves to the discussion of the paper it is a question of diet and alkalies. I agree with Dr. Joslin that the treatment of coma consists in its prevention. Instances of the control of coma are reported, but it is always fatal in the end. I think that the value of drugs lies in improving the resistance of the patient. May not assimilation in the diabetic be helped by drugs, as well as by rest and oxygen? Arsenic, glycerophosphates, nux vomica and other agents, may each help in individual cases. Though drugs may be of little service in treating the disease, yet I believe there is a place for them in the treatment of the patient.

Dr. Joslin, in closing: In regard to the use of drugs in diabetes, I agree with the speakers that occasionally remedies like bromides are of value in the treatment of the patient, but I never expect with drugs to permanently influence the glycosuria. If drugs are not prescribed, it means extra work for the doctor to be sure, because time must be spent with the patient in emphasising and explaining the part that diet plays in treatment. In the *British Medical Journal,* Vol. I., p. 544, 1903, is described a case that developed diabetic coma shortly after entering the hospital for treatment. This was apparently due to the change of diet. Such deaths are less common than formerly, and I doubt if many more are reported. Traveling often leads to coma, but this is unnecessary if the diet remains constant. In answer to Dr. Benedict's question, we have found that Squibbs's urea test may have 50 per cent. of error in diabetes. The relation of albumin to acid intoxication is not perfectly understood. Apparently, albumin, when it is burned in the body breaks up into a carbohydrate part and a part that is excreted in the urine. The oxidation of the former portion tends to prevent the development of acid intoxication. Exercise is a two-edged sword; it does well in some cases, and poorly in others. Of late the rest cure has been advocated for diabetes. It is a good thing for cases that are very much run down. Many cases of diabetes are fatal; on the other hand, cases frequently occur that do well under treatment. It is encouraging when a patient who excretes 6 per cent. of sugar in the urine, and is sugar free only on a diet containing less than one ounce of starch per day, improves so much that at the end of five years, five ounces of starch per day can be taken without glycosuria. The inside of bread, of course, contains less sugar than the crust, and yet diabetics are often advised to eat crusts. Three ounces of potato contain only as much sugar as one ounce of bread. The potato is bulkier, lasts longer, and is a good butter carrier, for which reasons it is a good food in these cases. Those interested in the problems of acid intoxication, will find them well discussed by Magnus Levy in the new edition of von Noorden's "Pathologie des Stoffwechsel," which is soon to appear.

Dr. Dewitt H. Sherman presented a paper entitled,

### THE PREMATURE INFANT.

*(Author's Abstract.)*

Dr. Dewitt H. Sherman, in his paper on The premature infant, reported the results of the incubators at the Children's Hospital. They have had twenty-nine cases, six of which died within twenty-four hours, and ten later. He reviewed the principal points of the lack of development of the premature. He dealt with the treatment under three heads: (1) maintenance of body temperature; (2) judicious feeding; (3) general care.

All the infants were out-patients. Thirteen had subnormal temperatures, 96° or less, and eleven died. One with a rectal temperature of 94° survived. His statistics showed the necessity of attending to the proper maintenance of temperature immediately after birth in order to prevent shock from chilling.

Under the heading of feeding, diluted breast milk is given almost immediately after admission without apparent ill-effects. The amount is small, the interval short, day and night. Plain breast milk is given as soon as tolerated. Some of the infants were too weak to suckle and were fed through the nose. Gastric lavage was not used, being too disturbing a procedure.

Under the heading general care he laid emphasis upon pure air of proper humidity; upon quiet and freedom from handling; upon anointing rather than bathing, except in so far as absolutely essential for cleanliness; upon the use of the fewest possible drugs, those used being mild cathartics and stimulants, and beef juice in the place of iron.

The smallest infant received weighed 1 pound 13 ounces. It died. The smallest surviving child weighed 2 pounds 11 ounces. One babe developed scurvy on breast milk. He claims that a premature infant can develop as well as one born at full term, and that the old notion that a seven months' infant is more liable to survive than an eight months' infant is groundless. He acknowledges the courtesy of Dr. Charles Summer Jones for the privilege of reporting all the cases.

DISCUSSION.

Dr. Charles Summer Jones: Indifferent care of the premature infant is not a new thing, but successful treatment is a new thing. The literature of the subject is meager at best and lacking in numerous important particulars. As incubator treatment is continued, much more will be known on these problems from year to year. As to artificial respiration in cyanosis, there are very good reasons for not resorting to it. Rotch reminds us that during intrauterine life the infant floats in the amniotic fluid, which is a provision for preventing jars and all such injuries as might follow handling. Then don't handle the premature infant. Artificial respiration will not save its life. Instead, use inhalations of oxygen, gentle counterirritation, an unusually humid atmosphere, slight medication by brandy and nux vomica; if these fail, artificial respiration will also fail, as was illustrated in the case of twins which I would like to cite.

In the premature there is a great tendency to muscular twitchings and convulsive seizures, due, probably to defective elimination of urea and to pressure on the brain. The head should lie on one side, to relieve pressure at the base of the head. The neck should be straight. The infant should have a generous quantity of sterilised water. Jaundice in the premature infant, though usually mild, is significant. Griffiths says that out of seventy-seven cases reported all but ten had a pronounced jaundice. In the Children's Hospital we have had a few cases of pronounced jaundice with regurgitation of bile from the mouth. The remedy may be accompanied by one twentieth of a grain of calomel once a day, to infants weighing three pounds. Jaundice is improved, also, by giving four or five minims of milk of magnesia with each feeding, and by increasing the humidity of the atmosphere. Details always count in these cases.

Dr. Sherman, in closing: I wish to emphasise the fact that in order to succeed with the incubator treatment we must have the infant immediately after birth, so as to get it while it is as near the normal temperature as possible. As to artificial respiration, my method is to place the infant in a warm mustard bath, its back resting on two hands of the operator, by which a slight flexion and extension is kept up,—simply enough to open and empty the lungs.

Dr. Charles G. Stockton: I move that, with Dr. Sherman's concurrence, we recommend to the council that a reprint of this most valuable paper be sent by the academy to every physician in Buffalo.

Dr. P. W. Van Peyma: I am impressed with Dr. Sherman's practical knowledge of his subject, and I heartily second the motion.

The motion was unanimously carried.

Dr. B. F. Herr, of Millersville, Pa., whose name appeared on the program, begged leave to present his paper at some future meeting of the academy.

The meeting adjourned at 11 p. m. Attendance, 68.

